# A method for electrophysiological characterization of hamster retinal ganglion cells using a high-density CMOS microelectrode array

**DOI:** 10.3389/fnins.2015.00360

**Published:** 2015-10-13

**Authors:** Ian L. Jones, Thomas L. Russell, Karl Farrow, Michele Fiscella, Felix Franke, Jan Müller, David Jäckel, Andreas Hierlemann

**Affiliations:** ^1^Bio Engineering Laboratory, Department of Biosystems Science and Engineering, ETH ZurichBasel, Switzerland; ^2^Visual Circuits Laboratory, Neuroelectronics Research FlandersLeuven, Belgium; ^3^NERF, ImecLeuven, Belgium; ^4^Department of Biology, KU LeuvenLeuven, Belgium

**Keywords:** retinal ganglion cells, RGC, MEA, CMOS, visual stimulation, cell classification, retina, sensory encoding

## Abstract

Knowledge of neuronal cell types in the mammalian retina is important for the understanding of human retinal disease and the advancement of sight-restoring technology, such as retinal prosthetic devices. A somewhat less utilized animal model for retinal research is the hamster, which has a visual system that is characterized by an area centralis and a wide visual field with a broad binocular component. The hamster retina is optimally suited for recording on the microelectrode array (MEA), because it intrinsically lies flat on the MEA surface and yields robust, large-amplitude signals. However, information in the literature about hamster retinal ganglion cell functional types is scarce. The goal of our work is to develop a method featuring a high-density (HD) complementary metal-oxide-semiconductor (CMOS) MEA technology along with a sequence of standardized visual stimuli in order to categorize ganglion cells in isolated Syrian Hamster (*Mesocricetus auratus*) retina. Since the HD-MEA is capable of recording at a higher spatial resolution than most MEA systems (17.5 μm electrode pitch), we were able to record from a large proportion of RGCs within a selected region. Secondly, we chose our stimuli so that they could be run during the experiment without intervention or computation steps. The visual stimulus set was designed to activate the receptive fields of most ganglion cells in parallel and to incorporate various visual features to which different cell types respond uniquely. Based on the ganglion cell responses, basic cell properties were determined: direction selectivity, speed tuning, width tuning, transience, and latency. These properties were clustered to identify ganglion cell types in the hamster retina. Ultimately, we recorded up to a cell density of 2780 cells/mm^2^ at 2 mm (42°) from the optic nerve head. Using five parameters extracted from the responses to visual stimuli, we obtained seven ganglion cell types.

## Introduction

Knowledge of cell types and corresponding functions in the retina provide insight into treatments for neurodegenerative diseases of the retina (Lagali et al., 2008; Bramall et al., 2010; Wright et al., 2010; Doroudchi et al., 2011). Many retinal diseases, such as age-related macular degeneration and retinitis pigmentosa, cause irreversible and progressive damage to the photoreceptors in the retina (Hartong et al., 2006); this group of cells initiates the sequence of events required for vision (Schmidt et al., 2008; Lorach et al., 2013). The photoreceptors convert visual stimuli into analog, graded-potential electrical signals that cascade through several layers of signal-processing cells to the retinal ganglion cell layer, which ultimately sends the visual signals to the brain through the optic nerve (Dowling, 2012). The ganglion cell layer has been found to contain 15–24 different types of ganglion cells (Masland, 2001; Brien et al., 2002; Rockhill et al., 2002; Dacey et al., 2003; Kong et al., 2005; Völgyl et al., 2009; Sanes and Masland, 2015). The development of methods to analyze and assess the precise physiological properties of ganglion cell types is essential for the understanding of retinal functionality and for prosthetic device technology (Chuang et al., 2014).

One method of functionally classifying the ganglion cells in the retina is to record the neuronal responses of ganglion cells while exposing them to visual stimuli. Research in electrophysiological characterization of the retina using visual stimuli dates back to 1938, when Hartline first demonstrated the existence of two types of frog ganglion cell types that responded to either bright or dark visual stimuli (Hartline, 1938). Since 1938, the proliferation and advancement of microelectrode-array (MEA) technology has made it possible to record from groups of cells; for a review of MEA platforms, see Obien et al. (2015). The MEA is an important tool that enables the recording of extracellular signals at high spatiotemporal resolution. MEAs have been used in retinal research for recording from significant fractions of cell populations (Meister et al., 1994; Devries and Baylor, 1997; Frechette et al., 2005; Farrow and Masland, 2011). However, extracting responses of individual ganglion cells is not trivial, as each electrode of the MEA will usually detect voltage signals generated by many ganglion cells, resulting in the mixing of the signals (Lewicki, 1998); therefore, spike sorting techniques must be applied to assign action potentials (or *spikes*) to the ganglion cells from which they originated. To maximize the accuracy of spike sorting, it is desirable to have the greatest possible number of electrodes relative to number of ganglion cells (Lewicki, 1998; Einevoll et al., 2012; Franke et al., 2012; Jäckel et al., 2012). When the MEA electrode density is too low within a given region of the retina, ganglion cells with low-amplitude signals, or those that are located in-between and/or distant from the electrodes are likely to be undetected or discarded by the spike sorting algorithm. Standard MEA systems are therefore somewhat limited in their ability to record from most of the ganglion cells of mammalian retinae; the ganglion cell density in the sections of hamster retina, for example, range up to approximately 3000 neurons/mm^2^, at a 2 mm eccentricity from the optic nerve head (Tiao and Blakemore, 1976).

To maximize the number of cells recorded in the ganglion cell layer, we used a complementary metal–oxide–semiconductor (CMOS) based high-density MEA (HD-MEA). The HD-MEA is ideal for retinal recordings due to its electrode density of 3150 electrodes/mm^2^. It has a total of 11,011 electrodes, and the selection of recording electrodes can be changed within milliseconds by means of a flexible switch matrix, which resides below the electrode array. The switch matrix capability allows for querying different regions of the retina within a 2 × 1.75 mm^2^ area. A low recording noise level of 2.4 μV_RMS_ in the action potential band (100 Hz–10 kHz) enables the recording of low-amplitude neuronal signals (Frey et al., 2010).

We used visual stimuli to stimulate ganglion cells in the hamster retina for the purpose of electrophysiological characterization: flashing squares were used for determining ON and OFF responses, and various moving bars were used for direction selectivity and spatiotemporal-dependent response component measurements. The stimuli were chosen to obtain the necessary responses from as many ganglion cells as possible during the limited time that the *ex-vivo* tissue was viable. Our goal was to maximize the throughput of our model by avoiding analysis and computation during the experiment that was previously necessary in other characterization studies (Carcieri et al., 2003; Zeck and Masland, 2007; Farrow and Masland, 2011).

## Materials and methods

### Tissue extraction and preparation

**Figure 1:**
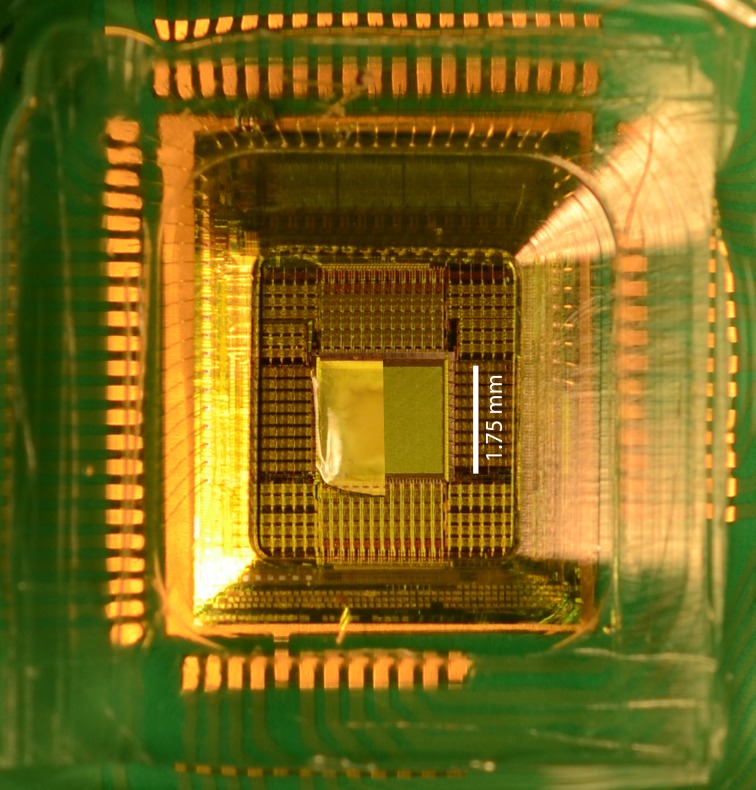
**HD-MEA chip**. Shown in the center of the chip is a sample of retina with a cutaway showing part of the microelectrode array (1.75 × 2 mm^2^) that lies underneath the retina piece; however, during an experiment, the MEA is fully covered by the retinal tissue. Around the MEA, the readout circuitry can be seen. Translucent epoxy packaging protects the periphery of the chip and the bond wires from liquid contact.

Eleven-week-old Syrian Hamsters/*Mesocricetus auratus* (Janvier Labs, France) were anesthetized and sacrificed under protocols that were approved by the Basel-City Veterinary office, in accordance with Swiss federal laws on animal welfare. Each hamster was kept in darkness for 10 min, anesthetized (Telazol 30 mg/kg, Xylazine 10 mg/kg) and decapitated. Retinae from both eyes were immediately removed under dim red light and immersed in Ames' Medium (8.8 g/L, supplemented with 1.9 g/L sodium bicarbonate: Sigma-Aldrich Chemie GmbH, Buchs SG, Switzerland), which was perfused with room-temperature Oxycarbon (PanGas AG, Dagmersellen, Switzerland) for at least 30 min before the optical stimuli sequence was started. To keep track of the anatomic orientation of the retina, the cornea was punctured just below the superior corneal limbus following removal of the eye from the animal, and a cut through the retinal tissue was made from the puncture location to the optic nerve head. The cornea was cut away, and the lens was extracted. The sclera was gently separated from the retinal tissue, and the remaining vitreal material was removed from the epiretinal surface; the retinal pigment epithelium was completely removed, as it would otherwise have obstructed the light path of the optical stimulus. A 1.5 × 1.5 mm^2^ section was cut from the superior nasal or superior temporal region, near the distal edge of the retina, and the tissue section was placed on the HD-MEA (see Figure 1). The retinal section was placed such that the ganglion cell layer (epiretinal side) was in contact with the HD-MEA surface, and the optical stimuli were focused directly onto the photoreceptor layer; this anatomical orientation was maintained for each experiment.

### Physiological apparatus

As shown in Figure 1, the HD-MEA was packaged by affixing a polycarbonate ring to it with epoxy, forming a well with a volume capacity of approximately 1 mL; the electrode array was located at the bottom of the well (Frey et al., 2007). The electrodes were coated with platinum black by electrodeposition so as to maximize the signal-to-noise ratio (lower electrode impedance) and to reduce photoelectric effects caused by the visual stimuli (Novak and Wheeler, 1986; Kim and Oh, 1996; Maher et al., 1999; Chang et al., 2000; Mathieson et al., 2004; Fiscella et al., 2012). A screw-mounted meshwork could be raised or lowered manually to apply sufficient pressure to hold the retinal tissue in place on the HD-MEA (retinal tissue on the MEA is shown in Figure 1). To maintain viability of the tissue, a gravity-flow system provided oxygenated Ames' Medium (see previous paragraph regarding physiologic solution) at a flow rate of 2.5 mL/min. The solution was heated to 35°C with a PH01 heated perfusion cannula (Multi Channel Systems MCS GmbH, Germany) and then directed with a plastic duct (length 1 cm; inner diameter 1.0 mm) onto the meshwork, adjacent to the subretinal (photoreceptor) side of the tissue, to oxygenate the retina as well as to flush metabolites produced by it. The perfusate was continually removed throughout the experiment by a suction needle, which was located at the surface of the solution bath.

### Light projection

Light stimuli were programmed in a Matlab (The Mathworks, Natick, MA)-integrated program, Psychtoolbox (http://psychtoolbox.org). The stimuli were sent to a LED projector (Acer K10), which had a refresh rate of 60 Hz, the output of which was spatially reduced by two camera lenses (Nikkor 60 mm 1:2.8 G ED, Nikon), dimmed in intensity by a 10x neutral density filter, deflected by 90° with a mirror (U-MBF3, Olympus), and focused with a 5x objective lens (LMPLFLN5X Olympus). The final image on the MEA surface was 1 × 1 mm^2^ and had the following intensity range: blue: 460 ± 15 nm; intensity of 2.0 × 10^13^ photons cm^−2^ s^−1^; green: 525 ± 23 nm; intensity of 3.3 × 10^13^ photons cm^−2^ s^−1^ as in Fiscella et al. (2012). The photopic intensity of the image could be controlled in 256 discrete steps. A single pixel of the image corresponded to an optical square of approximately 1.7 × 1.7 μm^2^ at the retinal surface. Only blue and green channels in the projector were used because Syrian hamsters are dichromats and are not sensitive to wavelengths in the red spectrum (Jacobs, 2002).

A video camera (Leica Camera AG) was connected to the microscope so that the HD-MEA surface could be viewed and monitored in real-time for focusing and spatial adjustment of the image on the retina. The HD-MEA was mounted on a computer-controlled moveable stage platform (Scientifica), which was moved within the x-y plane by a joystick for the purpose of aligning the center of the recording electrode configuration with the center of the projected image. This adjustment ensured that the selected recording electrodes were always in the same position relative to the projected optical stimuli.

Visual stimuli frame change timestamps were sent from the stimulation computer to a field-programmable gate array (FPGA) and recorded in parallel with the electrode data stream. This information made it possible to synchronize the projected stimuli with the recorded data in the *post-hoc* experimental analysis steps.

### Apparatus control

Four computers were used to control the apparatus. One computer running Red Hat Enterprise Linux (RHEL) was used for running the software for displaying the MEA output, for sending commands to the HD-MEA chip, and for recording data from the HD-MEA. A second computer running RHEL was used to run Psychtoolbox and send the visual stimulus frames to the projector. A third computer running RHEL was used to run Matlab for simple computations required during the experiment. Finally, a Windows XP computer displayed the real-time feed from the video camera; this computer also ran Scientifica® software to control the moveable stage along the x-, y-, and z-axes.

### Microelectrode array recordings

The data recorded on the HD-MEA were sampled at 20 kHz, and filtered on-chip, approximately between 0.3 Hz and 14 kHz. Prior to data post-processing, all of which was done in Matlab® (Natick, MA, USA), data were filtered with a 300 Hz high-pass filter and 8 kHz low-pass filter to reduce DC offset effects and high-frequency noise.

### Experiment preparation

Using a 1 mL pipettor tip (the end of the tip was cut off), the retinal segment was placed on the HD-MEA and aligned to the electrode array (see Figure 1) by removing liquid from around the retinal segment with a pipettor. The screw-mounted meshwork was lowered until it made contact with the subretinal surface. The tissue was immediately perfused with Ames' Medium, as described above, which was heated to 35°C with a TC01 temperature controller (Multichannel systems, Reutlingen, Germany). A 5-mm coverslip (Gerhard Menzel GmbH, Braunschweig, Germany) was placed between the microscope objective and the meshwork to maintain an optically aberration-free transition zone from the air to the liquid. The HD-MEA chip was plugged into the interfacing circuit board (the *Neurolizer*) to initiate communication with the HD-MEA. The position of the moveable stage, to which the Neurolizer was secured, was adjusted so that the center of the HD-MEA was visible in the center of the video monitoring screen. The image was then focused on the subretinal side of the retina by moving the objective lens. The retinal segment was allowed to acclimate to the MEA under a 50% contrast background for 20 min.

**Figure 2:**
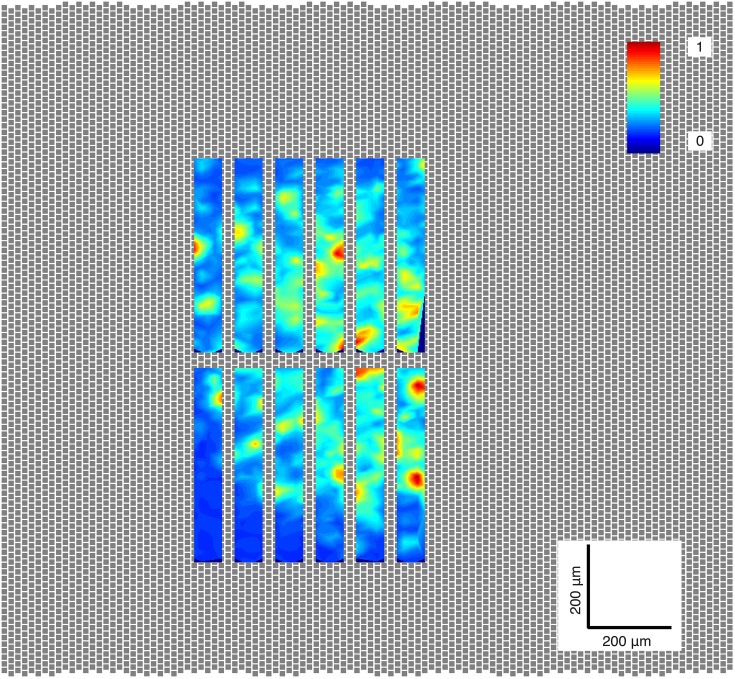
**Activity scan**. The location on the retina piece with the greatest number of ganglion cells was selected for each experiment. The electrodes of the MEA are shown as gray squares; the retina lies on top of these electrodes, 126 of which can be selected to simultaneously record ganglion cell activity. In this case, each rectangle shown in this image was generated by recording from a selected group, or *configuration*, of 126 electrodes. The colors represent the normalized peak-to-peak amplitude of the neurons found in each region.

To find the region of the retinal segment with the greatest number of active ganglion cells, a gray scale natural movie of mice moving in a cage was projected onto the center of the retinal segment. While the movie was being projected, a sequence of 12 non-overlapping recording electrode groups (or *configurations*) of 126 electrodes (area of 65 × 578 μm^2^; 0.0374 mm^2^) was used to record ganglion cell activity for 30 s each at 12 different regions of the retinal segment. All spikes produced by the ganglion cells within each recorded region were extracted by spike thresholding, and peak-to-peak amplitudes of these detected spikes were computed on all electrodes (Figure 2). The maximum peak-to-peak amplitude on each electrode was computed, and plotted on a heat map. The plot of the electrode configuration with the greatest number of local maxima (representing putative ganglion cells) was selected as the configuration to be used for the duration of the experiment.

### Light stimuli

The projected image was centered on the selected electrode region, and visual stimuli were run sequentially. Prior to each stimulus, a 50% contrast background was projected onto the retinal segment for a period of 5 min so that the cells could adapt to the mean projected photopic level. The stimuli were then shown sequentially, and a new data file was automatically started for each new stimulus.

#### Marching square over grid

The Marching Square Over Grid stimulus was used to find receptive field centers and to characterize ON-OFF response of each ganglion cell. The stimulus featured a 100 × 100 μm^2^ bright square that flashed at 81 non-overlapping pseudo-randomly-ordered locations over a 900 × 900 μm^2^ area. The stimulus presentation was 2 s in duration, and five repetitions were used. Complete stimulus set duration.

#### Narrow moving bars

Bright bars were used to determine the direction-selectivity properties of ganglion cells (Kanjhan and Sivyer, 2010). The bars moved along their longest axis, or *width* (width 1000 μm, length 500 μm), in eight radial equally-spaced directions with offsets of -250, 0, and 250 μm orthogonal to the vector of travel for each radial direction. The bars moved with a speed of 600–1200 μm/s (12.5 and 25 visual degrees/s). The purpose of the lateral offsets was to stimulate in all eight directions at every location on the selected region of retina. The bar closest to the receptive field center for each ganglion cell in each direction was then selected *post-hoc* to analyze each ganglion cell response. Complete stimulus set duration: 45 min.

#### Width test

Bright and dark rectangular bars of a range of widths (75, 150, 300, 600, 900 μm) and maximum length were used to obtain the spatial frequency response of the ganglion cells. The bars moved along the direction of the shortest axis in four equal radially-spaced directions. The bar movement speed was 150 and 900 μm/s (3 and 19 visual degrees/s). These speeds were selected to obtain responses from both ganglion cells that responded to fast and slow stimuli. Complete stimulus set duration: 75 min.

#### Speed test

Bright and dark rectangular bars (widths: 150, 600 μm) were shown at different speeds to assess the temporal frequency response of the ganglion cells. The bars moved along the direction of their shortest axis in four equal radially-spaced directions. The bars moved at speeds of 150, 300, 600, 900, 1200, and 1800 μm/s (3, 6, 13, 19, 25, and 38 visual degrees/s). Complete stimulus set duration: 60 min.

### Data analysis

#### Spike trains from raw data

**Figure 3:**
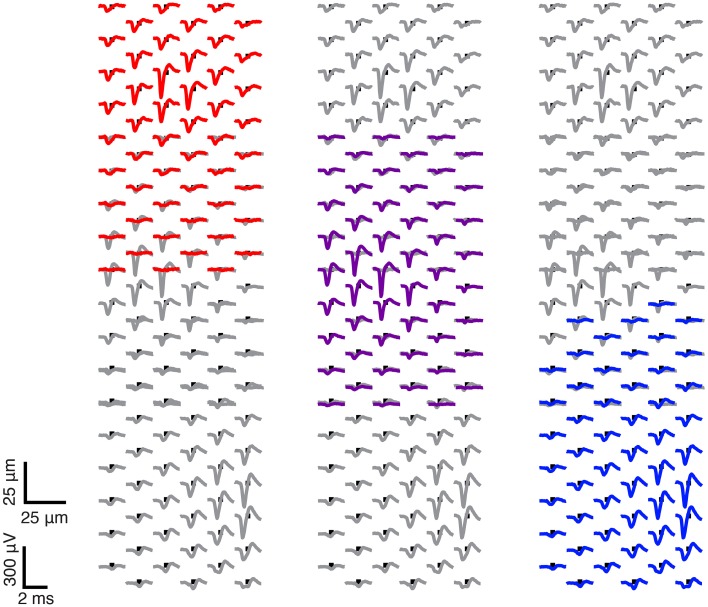
**Sample ganglion cells found in one electrode configuration**. Here is one exemplary electrode configuration, which has been used to record ganglion cells from the retina piece. Three sample spike-triggered average extracellular action potentials (STA-EAPs) of three ganglion cells are shown.

Signals from many ganglion cells were present on the majority of the array electrodes in a typical recording, therefore, data processing was necessary to obtain the spike trains from each neuron. Spikes were thresholded at 3.5 times the standard deviation of the baseline signal between spikes. Groups of seven recording electrodes were selected sequentially, and above-threshold spikes were demixed using principle component analysis (PCA; Lewicki, 1998; Jäckel et al., 2012). The demixed signals were used to generate a spike-triggered average extracellular action potential (STA-EAP), which served as a spike waveform template for each neuron (Figure 3). Finally, a template matching function scanned the entire recording to extract timestamps for each neuron (Franke et al., 2015).

#### Data quality

As a quality control check for the template matching algorithm, all multi-electrode templates generated in the previous step were compared in a pairwise fashion by computing the cross-correlation of the templates for each pair. Pairs that had a correlation coefficient of over 90% were determined to be duplicates; the multi-electrode templates of these pairs were visually inspected; and the number of spike time *violations*, or instances where a spike occurred within the refractory period of the ganglion cell, were computed. Spike time violations were not allowed to exceed 1% of the total number of spikes. Pairs that met the inspection and violation tolerance criteria were merged.

### Parameter calculation

#### Response latency

The latency index describes the delay between the appearance of the stimulus and the time at which the peak firing rate occurs. This index was computed by integrating over a window of 25 ms and measuring the length of time required to reach the maximum firing rate (Farrow and Masland, 2011). Units: seconds.

#### Transience index

The transience index indicates how long the ganglion cell continues to spike after the presentation of the stimulus. For each ganglion cell, the response to a flashing square was used to construct the post-stimulus time histogram (PSTH) with a duration of 1.5 s. The integral of the PSTH was normalized by dividing the area under the curve by 1.5 (Farrow and Masland, 2011). Range: 0 for an infinitely brief response; 1.0 for a continuous response during the 1.5 s post stimulus period.

#### ON-OFF bias index

The bias index (BI) describes the degree to which a ganglion cell responded to a bright or dark stimulus presentation. This index was computed using Equation (1), where *ON* is the spike count in response to a positive contrast stimulus, and *OFF* is the spike count in response to a negative contrast stimulus. Range: −1 for 100% OFF response; 0 for ON-OFF response; +1 for 100% ON response.

(1) $$\begin{array}{l} \text{BI}=\frac{\left(\text{ON}-\text{OFF}\right)}{\left(\text{ON }+\text{ OFF}\right)} \end{array}$$

#### Receptive field diameter

The receptive fields for all cells were mapped using the Marching Square Over Grid stimulus. The mean number of spikes in response to the flashing square were counted per location to determine where the ganglion was most sensitive to a light stimulus. To compute the mean receptive field area, the Marching Square Over Grid stimulus response map was upsampled nine times and thresholded at 33% of the maximum value. The pixel count within this area was divided by the total pixel count and scaled to the total projected area of the Marching Square Over Grid stimulus to provide the receptive field area, *A*. The mean diameter, *d* (units: μm), was computed as follows:

(2) $$\begin{array}{l} d=\sqrt{\frac{4A}{\pi }} \end{array}$$

#### Direction selectivity index

A direction selective ganglion cell responds maximally when the stimulus moves in a given direction across its receptive field. To compute this index, the mean firing rate during the presentation of bright bars moving in eight equally spaced radial directions was determined, and the vector average, *D*, was calculated, as shown in Equation (3). *v* is defined is the firing rate vector, and *r* is defined as the firing rate value. Range: 0 for no directional selectivity; 1 for a unidirectional response (Taylor and Vaney, 2002).

(3) $$\begin{array}{l} D=\left|\frac{\sum \overset{\to }{v_{i}}}{\sum r_{i}}\right| \end{array}$$

#### Preferred speed index

The preferred speed index indicates the preferred ganglion cell speed. During the Speed Test, moving bars were presented at different speeds. The mean peak firing response was computed at each speed, and the weighted mean of the responses was computed to obtain the peak response speed. The resulting *preferred speed* for each ganglion cell represented the speed at which the firing rate of the bar was greatest, across the parameter space of bar contrast and bar movement direction. Range: 0 for response to minimum speed; 1 for response to maximum speed.

#### Preferred width index

The preferred width index indicates the preferred ganglion cell width. During the Width Test, moving bars were presented at different widths. The mean peak firing response was computed at each width, and the weighted mean of the responses was computed to obtain the peak response width. The resulting *preferred width* for each ganglion cell represented the width at which the firing rate of the bar was greatest, across the parameter space of bar contrast and bar movement direction. Range: 0 for minimum width; 1 for response to maximum width.

### Parameter clustering with K-means

The following calculated indices or parameters extracted for each cell were clustered using k-means to sort the cells based on their electrophysiological responses: “Bias Index,” “Latency,” “Transience,” “DS-Index,” “Speed Index.” We ran this clustering algorithm with a preset *k*-value ranging from 4 to 25 and with 5000 repetitions to avoid local minima.

To assess the optimal number of clusters, we computed the Silhouette value of each member within the clusters and computed the mean Silhouette value for each group, according to Equation (4). The Silhouette value is a measure of separation between a given point and the other clusters of which it is not a member (Rousseeuw, 1987).

(4) $$\begin{array}{l} {\text{S}}_{\text{i}}=\frac{\left({\text{b}}_{\text{i}}-{\text{a}}_{\text{i}}\right)}{\max\left({\text{a}}_{\text{i}},{\text{b}}_{\text{i}}\right)} \end{array}$$

The formula for the silhouette value of one point, point *i*, within the dataset is shown above. b_i_ is the lowest mean dissimilarity (or distance) between the point *i* and all points in another cluster, of which *i* is not a member. a_i_ is the mean dissimilarity, or distance, compared to all other points in the same cluster. If b_i_ is much larger than a_i_, point *i* is well-separated from the other clusters and S_*i*_ will approach a value of one. If, however, the opposite is true, and b_*i*_ is much smaller than a_i_, point *i* is not well-separated from the other clusters and has probably been mis-assigned (Kaufman and Rousseeuw, 1990). We selected the number of cluster groups with the highest mean Silhouette value as the most well-separated number of clusters.

To confirm the ganglion cell type clusters obtained in the above steps, we computed the Fisher's linear discriminant for data points between all groups in a pair-wise manner. First, we computed the axis between the mean locations of each pair of clusters in 5-dimensional space (with each dimension corresponding to each parameter space for “Bias Index,” “Latency,” “Transience,” “DS-Index,” “Speed Index”). We then projected data points from each group onto this 1-dimensional axis and fitted a Gaussian function to the histogram of each group. To obtain the separation parameter, we used the following Equation (5):

(5) $$\begin{array}{r} \frac{d}{\sqrt{\left(\sigma_{1}\sigma_{2}\right)}} \end{array}$$

where *d* is the distance of the axis between the two group means in 5-dimensional space (μ_1_ and μ_2_, respectively), σ_1_ is the standard deviation of the Gaussian fit for the first group, and σ_2_ is the standard deviation of the Gaussian fit for the second group.

## Results

Two hundred sixty two ganglion cells that responded to visual stimuli were recorded in seven separate experiments. The ganglion cells were stimulated with the following visual stimuli: Narrow Moving Bars, Width Test, Speed Test, and Marching Square Over Grid (as described in Section Materials and Methods) in a procedure that required no spike sorting or detection of the receptive field during the experiment. Normalized descriptive parameters were extracted from the responses of each ganglion cell to the presented stimuli by quantifying the following properties: response to bright stimuli, dark stimuli or both (ON, OFF and ON-OFF responses, respectively); sensitivity to movement in a particular direction (direction selectivity (DS)); and spike train latency (brisk or sluggish) and duration (transient or sustained). The parameters were clustered to group ganglion cells into functionally-defined categories, resulting in the following ganglion cell types: ON brisk transient, OFF brisk transient, OFF DS sluggish sustained, ON-OFF brisk sustained, ON-OFF sluggish sustained, ON-OFF DS and ON DS (DS cells for multiple directions were found).

### Ganglion cell yield with HD-MEA

**Figure 4:**
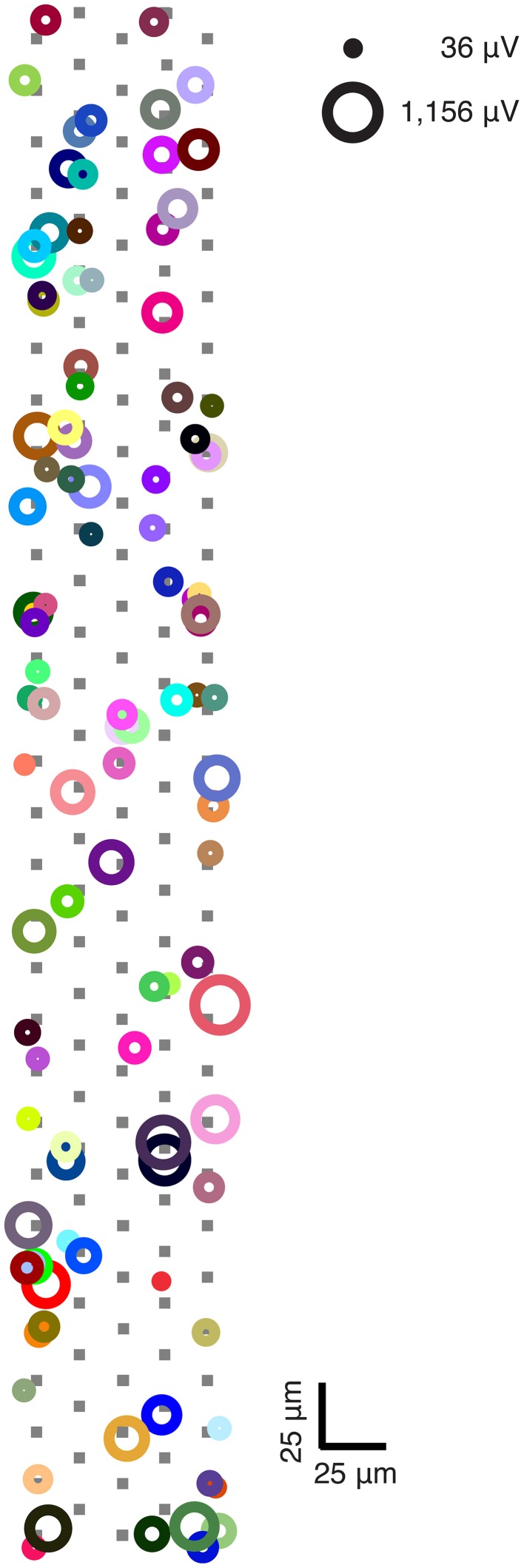
**Ganglion cell distribution and signal amplitudes within selected region of retina piece**. Each colored circle represents a unique spike-sorted ganglion cell that was recorded throughout one experiment and responded to at least one stimulus. The area of each circle represents each cell's peak amplitude. Locations of circles indicate the electrode where the peak amplitude of the spike-triggered average extracellular action potential (STA-EAP) were recorded. A ± 5 μm spatial jitter along the x- and y-axes was introduced into the plot to avoid circle overlaps. Overlaps occurred because the peaks of multiple ganglion cells were recorded on one electrode, which suggests that the axon initial segments of multiple neurons were closest to a given electrode. One electrode configuration was used to obtain this data: electrodes underlying cells are shown in filled gray squares. Note that not all electrodes shown in figure were used for recording.

One of our goals was to record a large number of ganglion cells per experiment to maximize the information obtained from a given region of the retina. An average of 55.3 ganglion cells ± 28.4 (range: 29–104 ganglion cells) per experiment were spike sorted, inside a mean area of 65 × 578 μm^2^ (0.0374 mm^2^) containing 126 recording electrodes; we recorded and spike sorted ganglion cell data up to a maximal ganglion cell density of 2780 cells/mm^2^ at approximately 2 mm (42°) from the optic nerve head to obtain the spike train for each detected ganglion cell (see Figure 4 for example of ganglion cell spatial distribution in one experiment). However, it should be noted that on average, only 68% of all cells responded to all visual stimuli.

### Responses to visual stimuli

#### Marching square over grid

**Figure 5:**
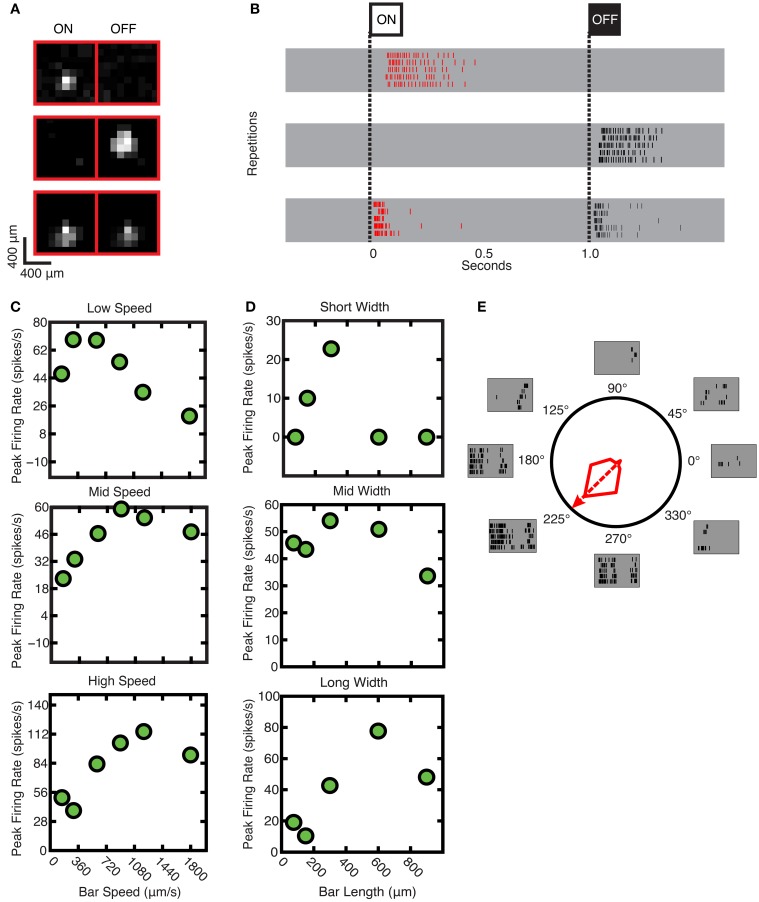
**Sample responses used to derive parameters. (A)** Receptive fields (from top to bottom) for ON, OFF, and ON-OFF ganglion cells. **(B)** Raster plots (from top to bottom) for the ganglion cell responses of ON, OFF, and ON-OFF ganglion cells. **(C)** Speed tuning of ganglion cells; from top to bottom: low, mid and high speed response characteristics. **(D)** Spatial tuning of ganglion cells; from top to bottom: short, mid, and long moving bar response characteristics. **(E)** Polar plot for response for typical DS cell, which responds most vigorously to movements in one direction, the *preferred direction*. The preferred direction of this specific cell is at 225°, and the *null direction* is at 45°.

Most ganglion cell types will respond to a marching square stimulus if it crosses the receptive field of that cell (Roska et al., 2006). Here, examples are shown of the responses of individual ganglion cells to flashing squares. In Figure 5A, the area over which each ganglion cell was responsive to light—the *receptive field*—is shown for these ganglion cells: from top to bottom, responses for ON, OFF, and ON-OFF ganglion cells are shown. In Figure 5B, the corresponding raster plots for ON, OFF, and ON-OFF cells in response to positive and negative contrast stimuli are shown. The uppermost gray band shows that the ganglion cell responded to the appearance of the bright square stimulus and was, therefore, determined to be an ON cell; the middle gray band shows that the ganglion cell responded to the dark square stimulus and therefore was an OFF cell; the bottom band shows a response to both bright and dark stimuli, indicating that an ON-OFF cell was found.

The *Speed Test* was used to characterize the ganglion cell response to different speeds of stimulus movement: this could also be termed the temporal response. As is shown in Figure 5C, some cells responded best to slower speeds (top panel), whereas others responded to intermediate (middle panel) or faster speeds (lower panel); (stimulus speed range 50–1800 μm/s). The tested range was selected based on previous experiments (Oyster, 1968; Wyatt and Daw, 1975; He and Levick, 2000; Sivyer et al., 2010), taking into account the anatomical dimensions of the hamster retina (Tiao and Blakemore, 1976).

The Width Test was used to characterize the spatial response: the ganglion cell response to different stimulus sizes, which is a function of the receptive field size. As is shown in Figure 5D, some cells responded best to narrow bars (top panel), while others responded to intermediately-sized bars (middle panel) or large sizes (lower panel); (stimulus bar width range was 75–900 μm).

Classic DS ganglion cells were also identified as shown in Figure 5E. This cell responded maximally to a moving bar in the cell's preferred direction (in this case, 225°), and exhibited no response when the bar was moved in the opposite, or “null” direction (Barlow and Hill, 1963).

### Parameters calculated

Descriptive parameters were extracted from the responses of the ganglion cells to the stimuli presented. The parameters were designed to represent generalized spatial, temporal and contrast sensitivity parameters for each ganglion cell across all ganglion cell types. Most parameters were normalized to a range of ± 1 so as to make direct comparisons, statistical computations and parameter clustering possible. When appropriate and possible, we used the parameter computation methods similar to those used by Farrow and Masland (2011).

**Figure 6:**
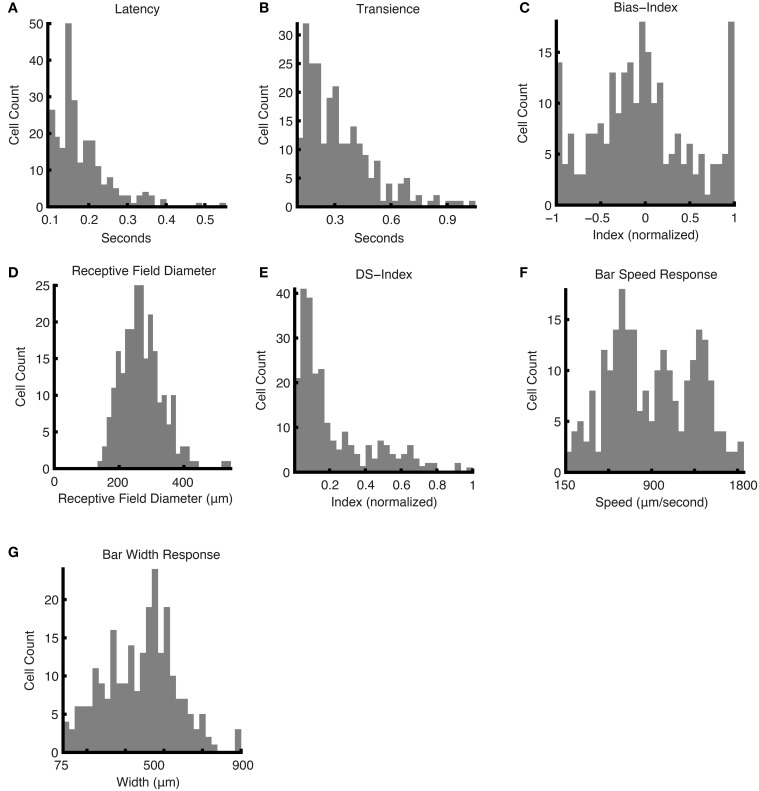
**Responses and parameters**. Histograms of responses and calculated parameters, showing the distributions for the entire dataset. The following were obtained using the responses to the Marching Square Over Grid stimulus: **(A)** latency values, **(B)** transience values, **(C)** bias indices, and **(D)** mean receptive field diameters. **(E)** The DS indices were computed using the Narrow Moving Bars stimulus in eight directions. **(F)** The bar speed response was computed from the responses to a moving bar at different speeds. **(G)** The bar width response was computed from moving bars with a range of widths.

The Marching Square Over Grid was primarily used to map out the receptive fields of the ganglion cells, but also provided temporal and contrast sensitivity response characteristics of the cells: see Figures 6A–D. To compute latency (Figure 6A), we measured how long a ganglion cell took to reach its maximum firing rate in response to a flashing square at the center of the receptive field: in the case of ON ganglion cells, the response to the bright stimulus was used, and in the case of OFF ganglion cells, the response to the dark stimulus was used. This latency measure peaked between 140 and 155 ms (21% of cells); 0.4% of cells exceeded 539 ms. Transience (Figure 6B), which measured the normalized area under the curve for this response and effectively the duration of the response, peaked between 87 and 108 ms (13% of cells), 0.4% of cells exceeded 679 ms. The ON-OFF Bias histogram (Figure 6C), shows a triphasic distribution at values of −1, 0, and 1, corresponding to OFF, ON-OFF, and ON cells respectively. The receptive field diameter had a monophasic distribution centered at 271.4 μm with a standard deviation of 64.3 μm (Figure 6D).

The Narrow Moving Bar stimulus was used to assess the direction selective (DS) component of the ganglion cells' responses, i.e., how sensitive the cell is to movement in a particular direction (Figure 6E). Assuming a threshold of 0.2 for a ganglion cell to be considered a DS cell (Rivlin-Etzion et al., 2012), we found that 35.1% of ganglion cells in our population were DS cells. This is consistent with the literature (Devries and Baylor, 1997; Rockhill et al., 2002).

Speed and Width Indices were computed during the Speed Test and Width Test, respectively, which involved, in the first case, varying the speed of the bar movement, and in the second case, varying the width of the bar. The Speed Index distribution appeared to be roughly trimodal: sensitivity peaks were found around the following parameter values: 0.30, 0.44, 0.60 (equivalent in speed units: 748, 1026, and 1345 μm/s; 15.7, 21.6; and 28.4 visual degrees/s; Figure 6F). The Width Index indicated a bimodal distribution, with parameter values at 0.34 and 0.56 (equivalent in width units: 307 and 503 μm; 6.5 and 10.6 visual degrees; Figure 6G).

### Clustering of parameters

The parameters were extracted to describe the visual response properties of the ganglion cells. After discarding neurons that failed to respond to all stimuli (125 were discarded from a total of 387 found ganglion cells), we normalized the receptive-field diameter, bar speed response and bar width response. We clustered the parameters with *k*-means from 4 to 25 and then computed the *mean silhouette values* for each value of *k* to determine the number of clusters that could be obtained from this dataset, which was found to be at *k* = 7, or seven clusters. This indicated that in our dataset, there were seven distinct groups of cell types that were reasonably separable from each other using five of the parameters (using all seven of the parameters did not reveal more clusters).

### Separability by center-to-center vector projection

**Figure 7:**
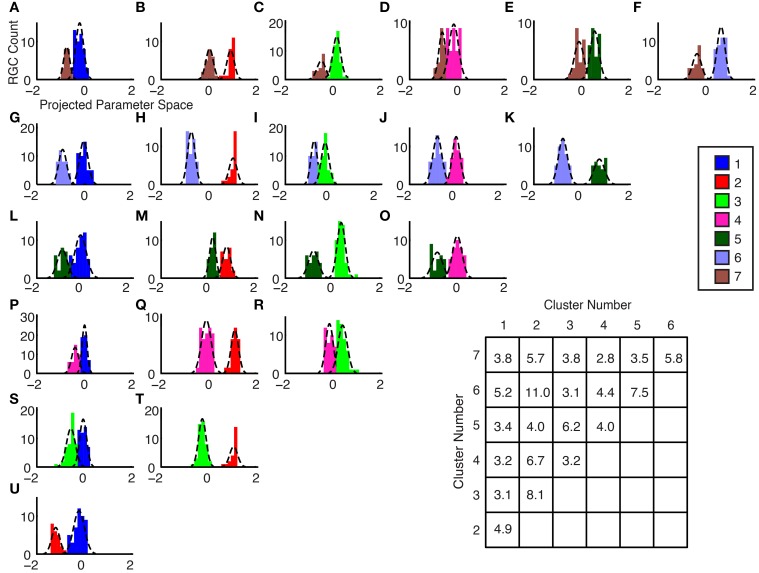
**(A–U)** Separability by Center-to-Center Vector Projection. Histograms and fitted Gaussians are shown for all possible pairwise combinations of the optimal number of clusters. The x-axis represents the axis between the 5-dimensional cluster mean locations for each of the groups. (inset table) Cluster Mean Separation Coefficient. The sigma error values from the Gaussian fits in Panels **(A–U)** were used to determine this coefficient (see Section Materials and Methods). A larger number indicates a greater separation between the groups. The y-axis labels indicate which group numbers were compared.

The degree of cluster separability is shown graphically by axis projections between pairwise comparisons of cluster group means in the 5-dimensional space: the parameter space consisted of one dimension for each of the parameters that were used for clustering; each ganglion cell therefore had unique coordinates in 5D space, according to its parameter values. The plots were generated as follows: each two cluster groups were selected for comparison, their mean locations in 5D space were computed, data points from each group were projected on a 1D axis spanning these two locations, and Gaussian curves were fitted to the data points of each cluster group. The curves for each group show the Gaussian curves of the pair of groups being compared. To illustrate: in Figure 7H, the separation discriminant between cluster #2 and #6 shows a clear separation, whereas in Figure 7S, the clusters #3 and #1 exhibit a lower degree of separation; this is quantified the inset table in Figure 7, where the Separation Coefficient has a value of 11.0 for the former case, and 3.1 for the latter. The separation coefficient for each comparison was computed (see Section Materials and Methods) and plotted in the table in Figure 7. The separability coefficient was equal or greater than 3.1 for all groups, which, given that the data has a uniform Gaussian distribution and approximately equal standard deviations for our datasets, would indicate that the two datasets are separated by three standard deviations.

### Parameter group characteristics

**Figure 8:**
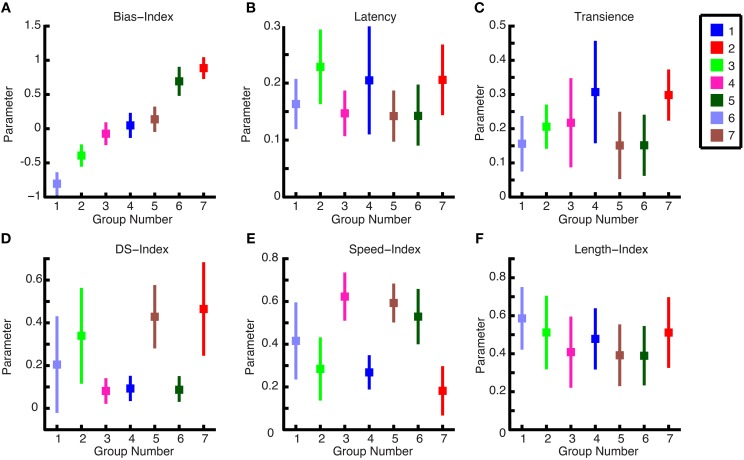
**Cluster characteristics. (A–F)** The mean parameter values for each ganglion cell are plotted against one another in a pairwise fashion to show the comparative values for each group. There are seven groups representing the cell types, and the ganglion cell members of each group are color-coded.

We computed the means of each group for each parameter to present the general characteristics for each group. For example, in Figure 8A, The ON-OFF Bias Index shows 2 OFF groups, 3 ON-OFF groups, and 2 ON groups. Furthermore, ON DS and ON-OFF DS cell types, which have been shown in the literature to exist for other mammals (Weng et al., 2005; Kanjhan and Sivyer, 2010), can be observed here. Namely, by examining Figures 8A,D, it can be seen that group 5 is an ON-OFF DS ganglion cell and group 7 is an ON ganglion cell. It can furthermore be observed, in Figure 8E, that group 5 responds at higher bar speeds, and group 7 to lower bar speeds, which is consistent with these two DS cell types (Wyatt and Daw, 1975). As is shown in this figure, the following cell types were found: OFF brisk transient, OFF sluggish transient, ON-OFF brisk transient, ON-OFF sluggish sustained, ON-OFF DS, ON brisk transient, ON DS.

### Members of clusters

**Figure 9:**
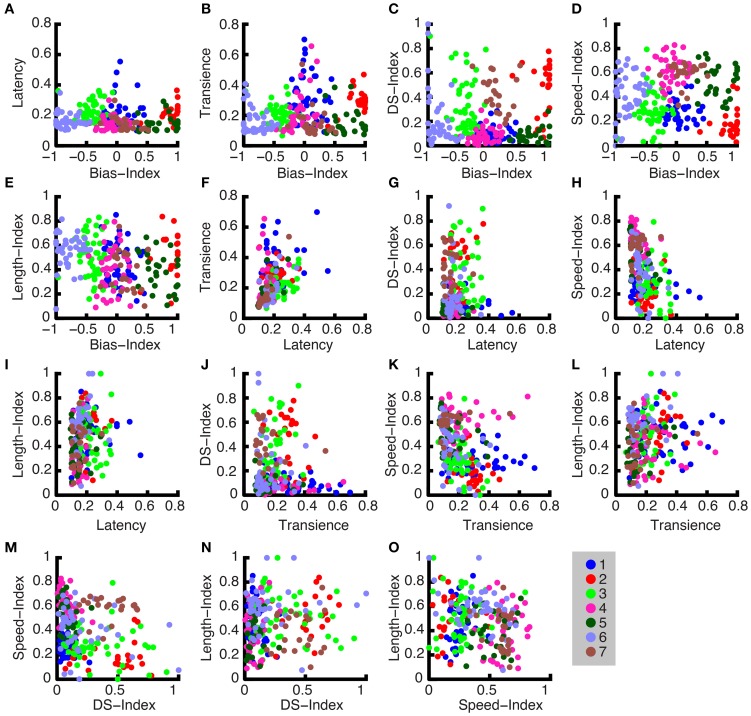
**Cluster members. (A–O)** All normalized parameters are plotted pairwise for all unique parameter combinations. The cluster groups, which show all members of each group, are indicated by their corresponding color code.

In Figure 9, the parameters are plotted against one another, and different colors are used to distinguish the clustered groups (note the group colors also correspond to those used in Figure 8). The groups are most clearly apparent when each parameter is plotted against the Bias-Index. However, in Figure 9E through Figure 9O, useful parameter distributions across the other dimensions are shown. For example, in Figure 9D, the group labeled with red circles has a bias index near 1 and a high DS index, which means that it is an ON DS cell.

### Confirmation of clustering

**Figure 10:**
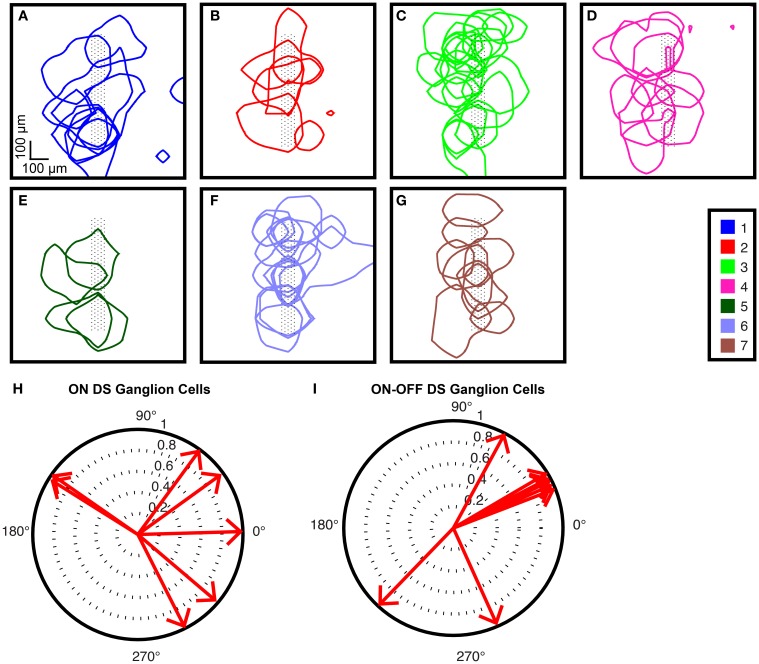
**Receptive field tiling (A–G)**. Receptive fields for all cells found in one experiment from one electrode configuration are shown. Cell cluster numbers are shown in the legend. Black dots represent electrodes under the recorded ganglion cells. The square size corresponds to the area over which the flashing squares were presented in order to map the receptive fields. Note that receptive fields extend beyond the electrode configuration, where corresponding ganglion cells were found. Direction-selective ganglion cells. Polar plots for the preferred directions of **(H)** ON DS ganglion cells and **(I)** ON-OFF DS ganglion cells that were recorded during this experiment.

To visually confirm that the clustering was successful, we used a standard method of plotting the receptive fields for each of the cell types found in one experiment (Field and Chichilnisky, 2007), as shown in Figures 10A–G. We then examined the responses of two cell types that were found among our clusters: ON DS ganglion cells and ON-OFF DS ganglion cells. Figure 10H shows the polar plots for ON DS ganglion cells (corresponding to cells in Figure 10B); responses for ON-OFF DS ganglion cells are shown in Figure 10I (corresponding to cells in Figure 10G).

## Discussion

We have shown that the combination of a CMOS HD-MEA with a series of visual stimuli provided an efficient method for investigating the response characteristics of ganglion cells in *ex-vivo* retinal tissue. This method was designed to maximally utilize the closely-spaced electrodes on the HD-MEA to record from as many ganglion cells as possible and to obtain accurate spike trains for each cell by spike sorting subcellular-resolution recordings. Additionally, we eliminated time-intensive spike sorting and computation of receptive fields during the experiment (these steps were all performed *post-hoc*) to stimulate the ganglion cells in an effective parallel manner.

### HD-MEA platform

Similar electrophysiological ganglion cell classification studies have been conducted with both the MEA and the patch clamp. However, we believe that the HD-MEA is the platform that provides the most advantages for these types of studies. While previous studies featured MEAs with electrode pitches of 25–100 μm electrodes (Carcieri et al., 2003; Segev et al., 2006; Zeck and Masland, 2007; Farrow and Masland, 2011), an electrode pitch of 17.5 μm enabled us to record signals from each ganglion cell on tens of electrodes, providing us with subcellular features that are essential for accurate spike sorting (Lewicki, 1998; Einevoll et al., 2012; Franke et al., 2012; Jäckel et al., 2012). A fine electrode pitch also made it possible to record cellular responses and a large number of parallel visual channels at a significantly higher ganglion cell density than would be feasible with a standard MEA device, providing information about ganglion cells closer to the center of the field of view. Finally, the HD-MEA is also better suited to large-scale ganglion cell identification or classification studies than is the patch clamp (Roska and Werblin, 2001; Weng et al., 2005), because the HD-MEA provides orders-of-magnitude greater throughput. Although the patch clamp method records signals that do not require sorting, the vastly higher throughput of the HD-MEA nonetheless justifies its use, in particular in view of the straightforward spike sorting as a consequence of the high electrode density.

Our recording electrode count of 126 also made it possible to record more data than was possible with the MEA devices featuring between 30 and 61 recording electrodes that were used in the studies mentioned above. The unique dynamic configurability of the recording electrodes enabled to always record from the most active part of the retinal sample. As mentioned in the Results Section, our maximum yield of spike-sorted ganglion cells of 2780 cells/mm^2^ at approximately 2 mm (42°) from the optic nerve head is approximately equal to what would be expected for cell density at that eccentricity in the hamster (Tiao and Blakemore, 1976).

### Visual stimuli

The visual stimuli were based on the work of Roska and Werblin (2001), Carcieri et al. (2003), and Farrow and Masland (2011): they were designed to include most basic visual characteristics (such as movement and contrast) to evoke the basic range of responses that would separate the greatest number of ganglion cell types, while being straightforward enough to analyze. However, while a common method of obtaining information about a ganglion cell's response to stimuli of varying sizes is to flash a spot of light of varying diameters (Van Wyk et al., 2006; Zeck and Masland, 2007; Farrow and Masland, 2011), this method requires locating the ganglion cell's receptive field during the experiment. To increase the efficiency of our recordings and avoid time-consuming spike sorting and signal processing, we chose to replace the flashing spots with moving bars of different widths. Moving gratings have been also been used to assess ganglion cell spatial response (Devries and Baylor, 1997; Sun et al., 2004), but we used moving bars instead to decrease the likelihood of any inhibitory effects that might suppress some ganglion cells' responses (Gollisch, 2013). Certain ganglion cell types have also been known to respond to variance in speed, most notably ON and ON-OFF DS cells (Wyatt and Daw, 1975; Grzywacz and Amthor, 2007); we therefore tested the responses of the cells to bars moving at varying speeds. Ganglion cell receptive field detection was examined by flashing a square randomly over the selected retinal region. While this method may have the disadvantage of possibly inhibiting responses in some cells (Troy, 2009), in our experience, we were able to obtain significantly more ganglion cell locations than with the random flickering checkerboard technique (Meister et al., 1994).

### Parameter space

The parameters that were extracted from the ganglion cell responses were meant to describe the ganglion cell behavior in a standardized manner, such that the cell type is to some degree identifiable, provided that there are sufficient parameters available (Olveczky et al., 2003; Segev et al., 2006; Zeck and Masland, 2007). As expected, the standard parameters of bias index, latency, transience and DS-index all provided response information that was useful for clustering cell types. The speed index was also found to vary amongst ganglion cell types, confirming our expectation that we would observe a cell type-depending speed response. The width index, however, did not provide much useful information, which indicates that in our studies, this index was not a very significant factor for defining cell type.

### Ganglion cell clustering

To sort the ganglion cell responses in parameter space, we chose a straightforward method for clustering: k-means. K-means is an effective data clustering method when cluster shapes are symmetrical (Kaufman and Rousseeuw, 1990); in the case of ganglion cell types, we expected that the parameters would cluster in a Gaussian distribution around a centroid, because ganglion cells of the same type would be likely to have similar parameter values across parameter space. K-means requires an a-priori cluster number, therefore, we used a range of *k*-values, from *k* = 4 to *k* = 25, which was selected according to the number of ganglion cells that we expected to find (Masland, 2001; Segev et al., 2006; Dowling, 2012).

The Fisher discriminant method to confirm the sorting parameters indicated that the least-well separated clusters had a discriminant value of at least 3.1 (table in Figure 7). As shown in Equation (4), the Fisher discriminant method is a metric of separation, based on the ratio of distance between the means (μ_1_ and μ_2_, respectively) of the Gaussian fits to the standard deviations of the Gaussian fits. Assuming a uniform Gaussian distribution and approximately equal standard deviations for our datasets, the Fisher discriminant is essentially equal to the number of standard deviations between the means of the compared datasets; the average Fisher discriminant across all pairwise comparisons in our dataset was 4.75.

It is known that most ganglion cell types tile the retina and that the receptive fields of a given type tend to have minimal overlap (Devries and Baylor, 1997; Field and Chichilnisky, 2007; Anishchenko et al., 2010). Thus, we tested our clustering for (i) coverage and (ii) overlaps. We did indeed see almost 100% coverage for all cell types: as shown in Figure 10, the electrode configurations under the neurons are to a large extent eclipsed by the receptive fields. However, because the number of ganglion cell types that we found was lower than the expected number for small mammals (see below for discussion), each cluster shown may have contained more than one cell type, which could explain why many receptive field overlaps are visible in these plots.

As an additional quality check of our clustering, we focused on two groups from one experiment: group 2 and group 7, which correspond to ON DS ganglion cells and ON-OFF DS ganglion cells, respectively. It has been reported that ON DS ganglion cells have a 3-lobed distribution in a polar plot, and ON-OFF DS ganglion cells have a 4-lobed distribution (Oyster and Barlow, 1967). In our data for ON DS ganglion cells (Figure 10H), we observed that the majority of the responding cells formed a 3-lobed distribution; the majority of the OFF DS cells (Figure 10I) formed a 4-lobed distribution. We therefore concluded that our clustering successfully identified these two cell types.

Several papers in the literature have attempted to determine the number of ganglion cells by computing parameters, but the literature is still not fully in agreement as to the absolute number of ganglion cell types (Lettvin et al., 1959; Grüsser-Cornehls and Himstedt, 1973; Cleland and Levick, 1974; Hochstein and Shapley, 1976; Caldwell and Daw, 1978; Stone, 1983; Carcieri et al., 2003; Schnitzer and Meister, 2003; Segev et al., 2006). Having found seven types of cells, we are in the lower range of the spectrum for expected number of cell types; however, we did find a similar number of types as in comparable studies (Lettvin et al., 1959; Grüsser-Cornehls and Himstedt, 1973; Cleland and Levick, 1974; Hochstein and Shapley, 1976; Caldwell and Daw, 1978; Stone, 1983; Carcieri et al., 2003; Segev et al., 2006). The explanation for our lower-than-expected number of ganglion cell types is that we did not explore the entire parameter space that would be necessary to find every cell type: for example, responses to luminance changes (Farrow and Masland, 2011), or a test of rod input to discern between ON cell subtypes (Deans et al., 2002). Regarding color vision: many smaller terrestrial species have a higher density of middle-wavelength-sensitive (M) cones in the superior part of the retina, and a higher density of short-wavelength-sensitive (S) in the inferior part (Yin et al., 2009). We did not test for such color variations and we only sampled retinal tissue from the superior portion of the retina.

### Outlook

This method has the potential for further development by increasing stimulus complexity to elicit responses from additional ganglion cell types, such as edge detectors, orientation-specific cells and uniformity detectors. The stimuli would need to be optimized in terms of stimulus repetitions, parameter resolution and parameter features to effectively utilize the duration of viability of the tissue. Use of an HD-MEA with more recording electrodes, such as that which has recently become available (Müller et al., 2015), would also increase the throughput of this method.

## Conclusion

We have demonstrated a method for rapidly recording from populations of ganglion cells in the retina and extracting a comparatively large amount of electrophysiological data from each ganglion cell in the population. This study sought to introduce a novel combination of an HD-MEA with basic visual stimuli to introduce an efficient categorization tool for ganglion cells. The HD-MEA has the advantage of allowing one to record virtually every ganglion cell within a selected region at high spatiotemporal resolution, such that the majority of ganglion cells are detected. The visual stimulus sequence was designed to make best use of the number of active recording channels available during the time that the retina preparation was viable and fully functional. As such, the sequence was intended to stimulate as many ganglion cells as possible (i) across as many dimensions as possible, (ii) in as short a time as possible, which was achieved by avoiding time-consuming spike sorting during the experiment so as to maximize our throughput.

### Conflict of interest statement

The authors declare that the research was conducted in the absence of any commercial or financial relationships that could be construed as a potential conflict of interest.
